# The Reaction toward Free Medicine

**Published:** 1915-02

**Authors:** 


**A Monthly Review of Medicine and Surgery**

EDITOR AND PUBLISHER, DR. A. L. BENEDICT, 228 Summer St., corner of Elmwood Ave., Buffalo, (Address for all communications. Please make personal and telephone calls before 1 P. M.)

Third, in age, on the western continent. Independent, but supports professional organization. News limited mainly to Western N. Y. and Penn.

Subscription $2.00 a year; $3.00 for two years in advance. Changes and errors in address or failure or duplication of delivery, should be reported immediately.

Yearly Volume 70. FEBRUARY, 1915. No. 7

### The Reaction Toward Free Medicine.

The Medical Society of the County of Erie, N. Y., has issued to its members an appeal for the general exercise of influence upon legislators, against the tendency to grant to special schools of practice of what may be termed under a broad definition, the healing art, without conforming to educational requirements. The President of the State Society, Dr. Grover W. Wende, has officially called the attention of the county societies to the fact that osteopaths, Christian Scientists, naturopaths and chiropractics came very near securing special exemption during the last session of the legislature, in the first two instances, only the governor's veto standing in the way.

The method of combatting the reactionary tendency proposed by the committee on Legislation under the chairmanship of Dr. Harvey W. Gaylord, is in keeping with modern business and ethical principles. It is persistent, repeated, general personal effort that secures results in forming and changing opinions.

Influence is not quite the right word to describe the method of securing proper legislation and of preventing improper legislation. Neither individually nor collectively, is the medical profession asking a favor for itself. The strength of its argument is not measured by its actual considerable numeric strength; nor is its claim in any way modified by the proportionate inconsiderable voting strength of the profession. It should be made plain that the word influence is here divested of its political significance and that the endeavor of the medical profession is solely to present facts, to secure legislation for the interests of the whole people, to speak as a profession only because its training and experience afford opportunities for clearer understanding.

Sometimes we become disgusted with the struggle, at different points and with different arms and ammunition, against the policy of guarding the health of the people by requiring that it be entrusted only to those specially trained. The policy, against that of free medicine, was apparently permanently established a quarter of a century ago or more, after thorough discussion of the various arguments, pro and contra. It has been justified by results to a degree beyond the most sanguine expectations. The death rate is 25% lower than formerly, the average span of life almost a decade longer, diseases formerly prevalent have become almost curiosities. Meantime, the average available work and source of income for physicians has steadily diminished, the requirement in preparatory and continuous study not directly profitable, has vastly increased and so has the responsibility of the physician to the state, even to the extent of his assuming duties not compensated for, requiring time and attention to detailed formalities, and involving heavy penalties for non-fulfillment. Looking at the matter in the narrowest and most personal way, the physician has not benefitted from the legislation which the profession has urged along the lines of medical control. On the one hand, the man who believes that organic disease can be prayed away, thought away or rubbed away, cannot be legislated into a source of income to scientific medicine. On the other hand, any method of treating disease which either does harm directly, or which allows the disease to progress by neglect of efficient treatment, ultimately tends to make work for the men who can "deliver the goods" or who can at the worst, express the definite opinion that the goods cannot be delivered at all.

It should be strongly impressed on the minds of all concerned that the medical profession is not opposing any manifestation of free medicine to restrict conpetition or to get business. The more latitude is given to men untrained in medical science and art, whether in the old fashioned way of allowing him to pick up experience and knowledge in a helter-skelter way, at the expense of his patients, or in the new fashioned way of allowing him to follow out a single hobby, more or less true but not generally applicable, the more serious work there will be for the man who is really competent.

It should be equally well understood that the control or non-control of medical practice is not a unique example of the question whether the law should require special preparation for particularly important and difficult fields of labor. There is a large number of persons holding peculiar views of what the law should be. These persons are not allowed either to frame laws according to their own conceptions or even to practice law in the ordinary sense of the term, until they have conformed to certain requirements involving the study of what the general consensus of legal opinion has crystalized into principles and codes of law. Having conformed to these requirements, they are free to impress their own peculiar views upon the people, to the extent of their influence. Very similar restrictions apply to plumbers, insurance companies and a number of other businesses. The medical profession, therefore, asks no special privilege for itself, not even a departure from precedent in framing special medical laws for the benefit of the people. All that has been demanded by the medical profession in laws or that can conceivably be demanded in the future, is that the individual must base his own personal expression of ideas upon a broad knowledge of what other men, who have devoted many life-times, throughout many centuries, to study and to acquire practical experience, have learned. Having conformed to this requirement, he is at perfect liberty to follow out his own ideas. It should be frankly admitted that there is a fallacy in this argument, but we think a perfectly legitimate one and one that will work to the advantage of the whole people. It is scarcely conceivable that any sane person, starting with the assumption, for instance, that disease is an error, that it has no real existence, will continue to hold this view in its extreme, literal sense, after having seen and studied the actual lesions of disease, macroscopically and microscopically, although he may apply the essential truth in his preconceived idea, to certain psychic aberrations. Nor, after having studied anatomy and physiology, and having had personal experience with perfectly tangible and visible evidence of certain chemic disturbances, is it conceivable that he will literally adhere to the universal applicability of purely mechanic methods in the treatment of disease. Analogously, it is scarcely conceivable that a man claiming the right to teach that the sun, planets and stars revolve directly about the earth, will continue to hold this view after having pursued a rigorous course in astronomy. But it does not strike us as an infringement on personal liberty to demand that, in medicine, as in law, plumbing, insurance, astronomy and various other matters, the person holding unusual views, contrary to those of the majority, should be compelled before putting them into practice, to hear the other and much larger side of the question.

An important element in educating legislative and popular opinion is to eliminate the conception of disease as something essentially mysterious and non-material. While there is much of which we do not and perhaps never may have a complete understanding, disease involves realities, tangible and visible things. It is perfectly demonstrable, for instance, that the definitely established infections present quite as real problems as the destruction of carpets by moths and problems indeed which must be solved by somewhat analogous methods. Without understanding the essentially material nature of various diseases, it is very easy to accept the idea that they may be treated either as non-existent or by methods which go wide of the mark. The most ardent Christian Scientist would scarcely hold that sediment in the carburetor could be disposed of by asserting its non-existence nor would an osteopath attempt to remedy the trouble by polishing the manifold. If the nature of calculus disease were comprehended as something equally definite, no sane man would advocate analogous methods of treatment and, on the other hand, he would appreciate that there might be an honest difference of opinion, either as to general policy or the best course in a particular case, between taking the machinery to pieces or some device for dissolving or forcing out the impediment, and between operations and medical treatment in the case of the pathologic analogue. Quite close analogies exist and are frequently alluded to in joke, between various phases of disease and plumbing problems. It would be well if such analogies were more generally and more seriously recognized and particularly on one account on which the individual physician is blamed and the general influence of the profession suffers. The plumber often informs us that a tank, faucet, pipe, closet bowl or other piece of work is worn out, was originally defective, has suffered from neglect, or has been damaged by accident or freezing, to such a degree that it is absolutely beyond repair; or that while it may be temporarily repaired, such a course is unwise and ultimately uneconomic. To a regretable and, at present, inevitable degree, a great deal of the physician's serious work is with just such cases. We are just reaching the point at which, like the plumber, we can rip out a worthless part and replace it with a new one. It is doubtful whether we shall ever be able to follow out this general method to anything like the degree possible in purely mechanic lines of industry but, while freely acknowledging our limitations, it is unjust to demand more than the period of development of the race has rendered possible.

Meantime, the medical profession asks legislation to prevent just such neglect and avoidable accident, just such prohibition of amateur tinkering and ignoring of tangible defects, as has been asked and received by plumbers. We challenge any one to show that in such legislation, any obstacle beyond the requirement of necessary training and skill has been placed in the way of anyone wishing to engage in the treatment of bodily disability and breakages or that, in any law passed or proposed at the request of the medical profession, there has been any attempt either to remove competition—with the qualification stated—or to fix the charges of physicians as interested parties, or to give them any other special privilege which does not inevitably attach to any legal requirement of special preparation and skill for any especially difficult and important calling. We may go farther than this in claiming good faith. No other profession, trade or industry, not even the government except in assigning counsel for defense in criminal cases, in a few forms of philanthropy, and in providing certain utilities on a co-operative basis, has so thoroughly insured the availability of service to all, irrespective of financial condition.

